# Supplementary material for: Schistosome infection among pregnant women in the rural highlands of Madagascar: A cross-sectional study calling for public health interventions in vulnerable populations
Source: PLoS Negl Trop Dis. 2024 Apr 16;18(4):e0011766. doi: 10.1371/journal.pntd.0011766 (PMC11051649; doi:10.1371/journal.pntd.0011766)
Supplement: S1 Table — (DOCX) [file pntd.0011766.s001.docx]

**Table S1. Comparison of sociodemographic characteristics of study participants and those excluded from the main analysis due to missing UCP-LF CAA test result**

|  | Included in the main analysis | | Excluded from the main analysis due to missing UCP-LF CAA test result | |  |
| --- | --- | --- | --- | --- | --- |
|  | n | % | n | % | p-value |
| **Age Group** |  |  |  |  | 0.1143 |
| 16-20 | 1,364 | 30.7 | 119 | 27.4 |  |
| 21-25 | 1,344 | 30.2 | 123 | 28.3 |  |
| 26-30 | 937 | 21.1 | 102 | 23.5 |  |
| 31-47 | 803 | 18.1 | 91 | 20.9 |  |
| **Recruitment region** |  |  |  |  | 0.0402 |
| Amoron'iMania | 2,092 | 47.0 | 165 | 37.9 |  |
| Bongolava | 598 | 13.4 | 58 | 13.3 |  |
| Itasy | 1,758 | 39.5 | 212 | 48.7 |  |
| **Urbanization** |  |  |  |  | 0.3356 |
| Rural | 3,133 | 70.4 | 326 | 74.9 |  |
| Peri-urban | 1,315 | 29.6 | 109 | 25.1 |  |
| **Education** |  |  |  |  | 0.1044 |
| No formal school | 166 | 3.7 | 23 | 5.3 |  |
| Primary | 2,218 | 49.9 | 231 | 53.1 |  |
| Secondary or higher | 2,064 | 46.4 | 181 | 41.6 |  |
| **Main contributor to family income** |  |  |  |  | 0.3447 |
| Participant | 104 | 2.3 | 14 | 3.2 |  |
| Other | 4,344 | 97.7 | 421 | 96.8 |  |
| **Occupation** |  |  |  |  | 0.7912 |
| Non-Farmer | 1,373 | 30.9 | 130 | 29.9 |  |
| Farmer | 3,073 | 69.1 | 305 | 70.1 |  |
