# Supplementary material for: Schistosome infection among pregnant women in the rural highlands of Madagascar: A cross-sectional study calling for public health interventions in vulnerable populations
Source: PLoS Negl Trop Dis. 2024 Apr 16;18(4):e0011766. doi: 10.1371/journal.pntd.0011766 (PMC11051649; doi:10.1371/journal.pntd.0011766)
Supplement: Attachment — Submitted filename: Reply to reviewers_v0.3.pdf [file pntd.0011766.s006.pdf]

BNITM
Bernhard Nocht Institute for Tropical Medicine

Bernhard Nocht Institute for Tropical Medicine
P.O. Box 50 06 40, 22706 Hamburg, Germany

Dr Daniela Fusco
Group leader

Bernhard-Nocht-Str. 74
20359 Hamburg | Germany
Tel +49 40 42818-504
Fax +49 40 42818-243
fusco@bnitm.de

Hamburg, 11.01.2024

To the Editorial Board of PLOS NTD

Dear Madams/Sirs of the Editorial Board, dear reviewers,

Thanks a lot for giving us the possibility to revise our manuscript and for the constructive comments which contributed to improve the quality of the paper.
We hope we correctly interpreted all reviewers' comments and we provide point by point reply to them. The figures have been uploaded and converted through PACE as per editorial requirements.
Please, if anything is unclear or consequence of misinterpretation of the revision, don't hesitate to come back to us for further clarifications.

Faithfully

Daniela Fusco, PhD

Bernhard Nocht Institute for Tropical Medicine • Foundation under Public Law • A member of the Leibniz Association
National Reference Centre for Tropical Pathogens
WHO Collaborating Centre for Arbovirus and Haemorrhagic Fever Reference and Research
Board of Directors: Prof. Dr. Jürgen May (Chair), Prof. Dr. Stephan Günther, Prof. Dr. Iris Bruchhaus, Birgit Müller
Chair of the Board of Trustees: State Counsellor Dr. Eva Gümbel
www.bnitm.de

Reviewer 1

Schistosome infection among pregnant women in the rural highlands of Madagascar: a Cross-sectional study calling for public health interventions in vulnerable populations

This paper reports a high prevalence of schistosomiasis among a large sample (over 4000) of pregnant women. This is an important topic because data on the prevalence of this neglected tropical disease highlights the need for integration of preventive schistosomiasis treatment into ANC packages in areas where schistosomiasis is highly endemic. I recommend the sharing of these findings with the schistosomiasis and maternal health community.

General comments

The current version of the paper reads quite well. However, I would like to make a few suggestions that could further improve the manuscript.

1. The aims and objectives seem to have been swapped, in my opinion. In general, a research aim should reflect the broad all-embracing goals of the research project. In other words, the aim should outline the general direction of the research and what it is trying to achieve. The objectives on the other hand should state how the study is going to answer the particular research question. In this case the questions are 1. What is the prevalence of schistosomiasis? 2. What are the factors that are associated with infection? /What are the risk factors for infection?

Reply: thanks a lot for the comment, we fully agree that we swapped aims and objectives. We adapted the concept both in the abstract (lines 29-31) and in the introduction as suggested in a further specific comment (lines 105 -109).

2. A comparison of the proportions of sociodemographic characteristics among women with and those without test results for schistosomiasis is a good way to dispel fears of underestimating or overestimating the prevalence of infection. If the two groups are similar it is highly unlikely that that the estimate would be different even though the missing results were available.

Reply: We are grateful to the reviewers for the suggestion and agree that the comparison of sociodemographic characteristics between women included and excluded from the study due to the absence of the test results is the first step to evaluate a risk of bias in the prevalence estimates.

We have compared sociodemographic characteristics between two groups using the chi-square test and observed no differences in the distribution of women by age group, education, urbanization, occupation, and contribution to the family income, however, the distribution of women by region was different with higher proportion of excluded in Itasy region. This finding motivated us to investigate further and perform the sensitivity analysis under the assumptions of all missing tests being positive and all missing tests being negative, which we already reported in the original version of the manuscript.

As suggested by the reviewer, we completed the presented sensitivity analysis with the comparison of sociodemographic characteristics of women, we incorporated this step in the description of the methods (lines 175-176) and results (line 255-256).

Finally, a supplementary table summarising the findings has been added in the supplementary materials as Table S1.

Specific comments are

Line 37, the first part of the sentence seems incomplete. I suggest rephrasing form “Among 4,448 women aged between 16 and 47 years, the majority of women (70.4%, 38, n = 3,133) resided in rural settings” to “Among 4,448 participating women aged between 16 and 47 years, the majority (70.4%, 38 n = 3,133) resided in rural settings.

Reply: the sentence has been adjusted as suggested (lines 41-42).

Line 79. Is the 1 at the end of the sentence the number for a recommendation in WHO document?

Reply: yes confirmed, it is the number of the recommendation. The 1 has been replaced with "number one" (now line 86) in order to avoid confusion.

Lines 97 101 (refer to comment # 1 above).
I suggest rephrasing the aim and objective as follows; “The aim of the study was to provide further evidence to support the adoption of public health strategies targeted at pregnant women for the management and control of schistosomiasis in endemic areas. The objectives of the study were to estimate the prevalence of schistosome infection and identify risk factors for infection among pregnant women in the rural highlands of Madagascar.

Reply: the sentences have been integrated as suggested.

Line 115. Exclusion criteria does not cover the opposite of inclusion criteria. In other words it should only state who among those that were included were to be excluded. When you say women aged 16 years and above were recruited it automatically means those below 16 were excluded. Exclusion criteria in this case should only include those having a history of congenital anemia, blood transfusion etc. meaning these were excluded from among those that met the inclusion criteria.

Reply: eligibility criteria have been adapted as suggested (line 125).

Line 137 Kindly write “LUMC” in full.

Reply: adjusted as suggested (lines 156-157).

Line 173. Were 5114 women recruited or that was the total number of women who were seen at the ANC during the study period? By implication using the word “recruited” means these were participants. Kindly rephrase for clarity. I would suggest using the word “screened” in place of “recruited” in line 173 and the chart.

Reply: here we want to clarify that the 5114 women were actually recruited for the study, those not included for the analysis were excluded for different reasons such as missing sample, failure of the testing to pass quality control or because after cleaning, not responding to inclusion criteria. Nevertheless, all the 5114 were actually recruited and included in the study (but not in the analysis, probably this is the nuance that makes the difference but this is specified in line 196) and followed the whole procedure of the study hence we think that the word recruited in this case is accurate but we are happy to modify in case, after clarifications, you still think that it is inaccurate.

Reviewer 2

General Remarks:
The manuscript titled "Schistosome Infection among Pregnant Women in the Rural Highlands of Madagascar: A Cross-Sectional Study Calling for Public Health Interventions in Vulnerable Populations" makes a commendable effort to address a significant knowledge gap. However, major revisions are recommended to enhance the manuscript's quality and impact by focusing on ensuring accuracy and adherence to best practices in scientific reporting. These considerations aim to contribute to the advancement of knowledge in this critical area. The dedication and commitment reflected in this research are appreciated, and anticipation surrounds the revised version, expecting it to significantly contribute to the scientific discourse in this field.
Specific Comments:

1. Species Specificity in Abstract and Methodology: The study lacks specificity regarding the identified Schistosome species in the abstract, methods, results, and discussion sections. Clear differentiation between S. mansoni and S. haematobium, along with explicit information on the prevalence of each, would strengthen the manuscript.

Reply: thanks a lot for the comment. We want to take the opportunity to clarify that there is no specific mention to the Schistosome spp in this paper since the method applied for the detection (UCP-LF-CAA) doesn't allow to differentiate species, since the CAA antigen is a genus specific antigen, as for example reported in this publication https://doi.org/10.4269/ajtmh.19-0819. More information can be additionally found in a recent mini-review, published by co-authors of this paper, in which they provide a general introduction to circulating antigen diagnostics with some general statements including the fact that these antigens are regurgitated by all *Schistosoma* species (https://www.frontiersin.org/articles/10.3389/fitd.2021.722438/full). For CAA this is highlighted further, with specific references indicating that the test works well for various different species (*'CAA can be detected in urine and serum via the Up-Converting reporter Particle technology based, Lateral Flow (UCP-LF) CAA test (44). The UCP-LF CAA test has been evaluated in various endemic and non-endemic settings and has demonstrated high specificity and sensitivity for the detection of all human species including hybrids (31, 34, 35, 37, 44–52*). In order to make this clearer to all readers, we added a sentence to clarify the readout of the test in the methods section, lines 151 and 152.

2. Age Association in Discussion Section: The observed significant association with age requires a more robust justification. Establishing the novelty of this association by drawing comparisons with related studies or providing a more detailed rationale is recommended.

Reply: clarifications, rationale and reference to compare and justify the finding have been added in the discussion, lines 289-295. We also want to clarify that other studies have identified age as a possible factor associated to schistosome infection or at least trends have been observed pointing in this direction (added as references 25-28 of the version 0.8 of the manuscript). We want to highlight that we don't find surprising this finding and this is somehow "a novel finding" mostly due to the fact that studies in adult populations are scarce and the main focus of schistosomiasis has been traditionally on school aged children. As we show in the paper Gruniger et al, 2023 (reference 21 of the manuscript) an association of the infection with farming can be observed. In fact, we also observe that high rate of infection among adults can be found in "working age" ranges supporting the hypothesis that schistosomiasis could be considered not only a disease of childhood but also an

occupational disease. In the present study we don't observe this association with statistical significance and probably this is due to the fact that during pregnancy, even if conventionally farmers, women don't work as much as they would if not pregnant hence the exposure is lower than usual.

3. Comparison with Control Group: The absence of a comparison with a control group is notable. Including a control group is crucial for a comprehensive interpretation of the observed prevalence among pregnant women. Addressing this deficiency in the study design and analysis is recommended.

Reply: thanks a lot for this comment and we hope we interpreted this correctly. The present study has a cross-sectional design, hence by design we did not include a control group. We acknowledge about the importance of comparing this group with i.e. the general population. Unfortunately, data in Madagascar are scarce and mostly reported in school aged children as assessments for Mass Drug Administration interventions. A recent study published by our group (Gruniger et al, 2023 10.1186/s40249-023-01094-z) is the first study in Madagascar assessing prevalence in adults, though the detection method is different to allow a proper comparison. Anyway, we added a sentence in the discussion, lines 268-270 in order to give some additional context to the finding.

4. Methodology Clarity: The methodology could benefit from greater clarity, particularly in providing a step-by-step account of the study procedures. Explicitly stating what was done during the study and ensuring alignment with the presentation of results and subsequent discussions will enhance transparency. The choice of diagnostic method raises a concern, as the POC-CCA test is designed for S. mansoni, while the UCP-LF test detects S. haematobium. Clarifying the Schistosome species identified and explaining the rationale behind the choice of test would enhance the manuscript.

Reply: thanks a lot for this comment. Considering that the recruitment and its details were described in the publication Fusco et al, 2022 (ref. 16 of the present manuscript) we considered redundant to specify them also in this publication. Regarding the use of the tests, to follow your suggestion, we specified in lines 118-120 that the POC-CCA results were not used for this specific study. The choice for not using the POC-CCA results for this specific study is due to the fact the test performances in pregnant women have not been sufficiently assessed and since the paper Hosmana et al, 2020 (https://doi.org/10.1186/s40249-020-00733-z) reports lack of specificity of the test due to cross-reactivity to unspecified biomarkers, we preferred to opt for the more precise test UCP-LF-CAA for the scope of the prevalence assessment. As highlighted in the reply of the comment number 1, the test used doesn't allow to discriminate between species hence to be accurate we cannot report species in the present publication. In addition, UCP-LF-CAA doesn't show different performances depending on the species while the POC-CCA seems to work better for *S. mansoni* than for *S. haematobium* mainly because higher amounts of CCA excreted/detectable in urine in case of an *S. mansoni* infection as opposed to *S. haematobium*. These last data are still unpublished but the manuscript is in preparation from the university of Leiden, developer of both tests and co-authors of this paper.

5. Repetition and Clarity in Abstract: Repetition is observed in the abstract and lines 84-85. Rephrasing to eliminate redundancy will contribute to overall clarity.

Reply: thanks a lot for the remark. The abstract has been modified (line 26) in order to be less redundant with the introduction.

6. Title Consideration: Given the need for specificity regarding the identified Schistosome species, consider revising the title to reflect the particular species observed. A more precise title will enhance the manuscript's clarity and relevance.

Reply: given the explanations about the species and the test used, we think that species shouldn't appear in the title. We hope that our clarifications are sufficient, otherwise we will be happy to reconsider.

7. Elaboration on Feasibility and Logistics: The discussion on the feasibility and logistics of implementing preventive measures in Antenatal Care Services could be more detailed. Providing insights into practical integration, addressing potential challenges, and offering implementation process details would strengthen this aspect.

Reply: implementation suggestions have been added in the discussion in lines 334-337.

8. Typo in Author Summary (Line 47): A minor typographical error is noted in the Author Summary section (line 47), where "Schistosomiasis" is misspelled as "Schistosomias."

Reply: checked and validated.

9. Broader Implications for Public Health: Emphasizing the broader implications of your study findings for public health policy and practice is encouraged. Explicitly discussing how your research contributes to shaping policies, interventions, and practices, with a focus on broader public health impact, will enhance the manuscript's significance.

Reply: the conclusions have been adapted according to the comments in order to emphasize the public health relevance of our findings in lines 371-377.

Thank you.
