# Supplementary material for: Schistosome infection among pregnant women in the rural highlands of Madagascar: A cross-sectional study calling for public health interventions in vulnerable populations
Source: PLoS Negl Trop Dis. 2024 Apr 16;18(4):e0011766. doi: 10.1371/journal.pntd.0011766 (PMC11051649; doi:10.1371/journal.pntd.0011766)
Supplement: Attachment — Submitted filename: Second Revision PNTD-D-23-01372_reviewer Comments 09 02 2024.pdf [file pntd.0011766.s007.pdf]

PNTD-D-23-01372_reviewer Comments

General Remarks:

The manuscript titled "Schistosome Infection among Pregnant Women in the Rural Highlands of Madagascar: A Cross-Sectional Study Calling for Public Health Interventions in Vulnerable Populations" makes a commendable effort to address a significant knowledge gap. The manuscript has improved a lot. However, the authors need to make minor corrections as suggested below to enhance the manuscript's quality and impact by focusing on ensuring accuracy and adherence to best practices in scientific reporting. A few grammatical errors were also seen.

Abstract

Results: Among 4,448 participating women aged between 16 and 47 years, the majority 39 (70.4%, 38(not sure what this means) n = 3,133) resided in rural settings. Overall, the prevalence of schistosome infection was 55.9% (…./4,448) ((aPR missing CI 95%: 53.3-58.5) increasing with age. The authors should present the results very well.

Line 44: 44 supports the consideration of preventive schistosomiasis treatment in ANCs of the Malagasy (wrongly spelt).

Conclusion (The authors should revisit their conclusion in the discussion section and use it to rewrite this part.) There is a need for better alignment between the discussion and conclusion sections.

Methodology

Line 119: Excluded from the study were women having a history of congenital anaemia, blood transfusion, epileptic or convulsive episodes, (include **or**) fever at the day of recruitment (The authors should include the rationale for the exclusion)

Line 127: A urine sample was collected from each participant and stored on-site at room temperature for 128 a maximum of seven days. (The authors should consider stating why the urine sample was stored for a maximum of seven days)

Line 139: Post-testing quality control was performed by Leiden University Medical Center (LUMC). (Not clear who performed/ carried out the Post-testing quality control and what percentage were used. Or were all the samples subjected to it?

Line 200: The authors should space this (CI95%: 53.3 - 58.5) so that it will be clear. Of those tested positive on-site, 99.9% (…/y) accepted treatment with PZQ. The actual proportion should be included.

Lines 204-207: The proportion of positive for schistosome infection also varied by region,being the lowest in Bongolava 50.3% (CI95%: 46.4 - 54.3), and the highest in Itasy 57.6%(CI95%: 53.7 - 61.3). Moreover, we report a 54.4% (CI95%: 52.1 - 56.6) schistosome positivity among women living in rural settings. (The authors should consider including the statistical significance based on location considering the fact that emphasis were made about the rural communities. This should also be reflected in the abstract and discussion too). Adding information about the statistical significance based on location, particularly in rural communities, would provide valuable context and insight into the regional variations in schistosome infection

prevalence. This addition would enhance the interpretation of the results and provide a more comprehensive understanding of the impact of location on infection rates.

Line 230: Uptake of antimalaria preventive treatment (aPR=0.85, CI 95%: 0.77-0.95), was associated with a decreased prevalence of schistosome infection. (The authors should include this information in the abstract.

Sensitivity analysis

Line 240: Under the assumption of participants with missing UCP-LF CAA test results being all positive, the prevalence estimate was 59.8% (CI 95%: 57.3 - 62.3). (The authors should state how the assumption was reached statistically and by what percentage. If it is possible, include an authority to make this more scientific).

Line 243: The aPR estimates were similar to ones from the main analysis under both assumptions, with the differences from the original estimates ranging from 0% to 6.3% (less than 10% for all variables) (How did the authors arrive at this range? Not clear. Please include the method used to arrive at this.). There is need for clarity on how the range of differences from the original estimates was calculated.

Discussion

Lines 278-279: Additionally, the results of multivariable regression analysis showed that women previously treated with PZQ did not have a different chance to be actively infected with schistosomes. (The authors should include the rationale such as that the treatment with praziquantel does not confer immunity and this shows also that the behavioural or environmental factors persist).

Lines 288-300: The authors did not carry out a comparative test between microscopy and the urine-based upconverting reporter particle, lateral flow (UCP-LF) test detecting circulating anodic antigen to make this type of assertion. They should focus more on the main objective of this study and suggest this aspect as a limitation to the study. A limitation regarding the lack of comparative testing between microscopy and the UCP-LF test should be acknowledged and discussed as even the method used here by the authors has its own challenges.

Lines 349-359: Based on the findings of the present study, we strongly advocate for adapting schistosomiasis programs (**with the aim of integrating** replace with **to integrate**) preventive schistosomiasis treatment in ANCs of highly endemic contexts. This would contribute to both the WHO and SDGs agenda overall to improving the well-being of women and consequently breaking the vicious cycle of poverty perpetuated by Schistosomiasis. (The authors should capture this in the conclusion section of the abstract).

Thank you.
