# Supplementary material for: Schistosome infection among pregnant women in the rural highlands of Madagascar: A cross-sectional study calling for public health interventions in vulnerable populations
Source: PLoS Negl Trop Dis. 2024 Apr 16;18(4):e0011766. doi: 10.1371/journal.pntd.0011766 (PMC11051649; doi:10.1371/journal.pntd.0011766)
Supplement: Attachment — Submitted filename: Reply to reviewers_Second Round_v0.1.pdf [file pntd.0011766.s008.pdf]

BNITM
Bernhard Nocht Institute for Tropical Medicine

Bernhard Nocht Institute for Tropical Medicine
P.O. Box 50 06 40, 22706 Hamburg, Germany

Dr Daniela Fusco
Group leader

Bernhard-Nocht-Str. 74
20359 Hamburg | Germany
Tel +49 40 42818-504
Fax +49 40 42818-243
fusco@bnitm.de

Hamburg, 22.02.2024

To the Editorial Board of PLOS NTD

Dear Madams/Sirs of the Editorial Board, dear reviewers,

Thanks a lot for highlighting the improvement of the manuscript and we hope that the revisions included in the current version will be satisfactory to move forward with the publication
We hope we correctly interpreted all reviewers' comments and we provide point by point reply to them.
Please, if anything is unclear or consequence of misinterpretation of the revision, don't hesitate to come back to us for further clarifications.

Faithfully

Daniela Fusco, PhD

Bernhard Nocht Institute for Tropical Medicine • Foundation under Public Law • A member of the Leibniz Association
National Reference Centre for Tropical Pathogens
WHO Collaborating Centre for Arbovirus and Haemorrhagic Fever Reference and Research
Board of Directors: Prof. Dr. Jürgen May (Chair), Prof. Dr. Stephan Günther, Prof. Dr. Iris Bruchhaus, Birgit Müller
Chair of the Board of Trustees: State Counsellor Dr. Eva Gümbel
www.bnitm.de

PNTD-D-23-01372_reviewer Comments

General Remarks:
The manuscript titled "Schistosome Infection among Pregnant Women in the Rural Highlands of Madagascar: A Cross-Sectional Study Calling for Public Health Interventions in Vulnerable Populations" makes a commendable effort to address a significant knowledge gap. The manuscript has improved a lot. However, the authors need to make minor corrections as suggested below to enhance the manuscript's quality and impact by focusing on ensuring accuracy and adherence to best practices in scientific reporting. A few grammatical errors were also seen.

Abstract
Results:
Among 4,448 participating women aged between 16 and 47 years, the majority (70.4%, (not sure what this means) n = 3,133) resided in rural settings. Overall, the prevalence of schistosome infection was 55.9% (…./4,448) ((aPR missing CI 95%: 53.3-58.5) increasing with age. The authors should present the results very well.

R: thanks a lot for the comments that we hope to be able to address.
- the majority (70.4%, (not sure what this means) n = 3,133): a total of 3,133 women which is the 70.4% of the total population was resident in a rural setting.
- 55.9% (…./4,448): included in the text now line 40
- ((aPR missing: it is not missing but it doesn't apply to this result since here we are not comparing groups but reporting the overall prevalence.

Line 44: 44 supports the consideration of preventive schistosomiasis treatment in ANCs of the Malagasy (wrongly spelt).

R: We checked, but we couldn't find a spelling error - in case it is there can please the reviewer specify the exact word that is wrongly spelled?

Conclusion (The authors should revisit their conclusion in the discussion section and use it to rewrite this part.) There is a need for better alignment between the discussion and conclusion sections.

R: Adapted as requested in lines 48-51

Methodology

Line 119: Excluded from the study were women having a history of congenital anaemia, blood transfusion, epileptic or convulsive episodes, (include or) fever at the day of recruitment (The authors should include the rationale for the exclusion)

R: rationale for exclusion criteria has been included in lines 127-130

Line 127: A urine sample was collected from each participant and stored on-site at room temperature for 128 a maximum of seven days. (The authors should consider stating why the urine sample was stored for a maximum of seven days)

R: a sentence was added on lines 141 and 142. To clarify, given the experimental nature of the test adopted, there is no sufficient information regarding the stability of the samples at room temperature,

hence this limit was set arbitrarily by the reference laboratory at the Leiden University Medical Center, based on experience of previous studies.

Line 139: Post-testing quality control was performed by Leiden University Medical Center (LUMC). (Not clear who performed/ carried out the Post-testing quality control and what percentage were used. Or were all the samples subjected to it?

R: clarifications were added in line 154-155.

Line 200: The authors should space this (CI95%: 53.3 - 58.5) so that it will be clear. Of those tested positive on-site, 99.9% (…/y) accepted treatment with PZQ. The actual proportion should be included.

R: adjusted in the text, line 216

Lines 204-207: The proportion of positive for schistosome infection also varied by region, being the lowest in Bongolava 50.3% (CI95%: 46.4 - 54.3), and the highest in Itasy 57.6%(CI95%: 53.7 - 61.3). Moreover, we report a 54.4% (CI95%: 52.1 - 56.6) schistosome positivity among women living in rural settings. (The authors should consider including the statistical significance based on location considering the fact that emphasis were made about the rural communities. This should also be reflected in the abstract and discussion too). Adding information about the statistical significance based on location, particularly in rural communities, would provide valuable context and insight into the regional variations in schistosome infection prevalence. This addition would enhance the interpretation of the results and provide a more comprehensive understanding of the impact of location on infection rates.

R: thanks a lot for the comment. We would like to highlight that for this specific result we don't report significance as this is not part of the adjusted Prevalence Ratio calculation (modelling analysis) for which instead significance is reported. Additionally, we didn't identify any significance difference for this specific factor. As follows from the results of the Poisson regression model presented in the results section, the differences in the prevalence of schistosome infection by region, urbanization, education, contribution to the family income, previous treatment with PZQ, uptake of iron/folate supplement and anti-helminth medication were not statistically significant. To clarify this point we reformulated the result section, to avoid statements that could mislead readers about the statistical significance of the differences in the estimates (lines 220-224).

Line 230: Uptake of antimalaria preventive treatment (aPR=0.85, CI 95%: 0.77-0.95), was associated with a decreased prevalence of schistosome infection. (The authors should include this information in the abstract.

R: The element has been added in the abstract as requested, lines 41-43.

Sensitivity analysis

Line 240: Under the assumption of participants with missing UCP-LF CAA test results being all positive, the prevalence estimate was 59.8% (CI 95%: 57.3 - 62.3). (The authors should state how the assumption was reached statistically and by what percentage. If it is possible, include an authority to make this more scientific).

R: During the study we were not able to collect and process urine samples for a part of the participants (n=435). Those participants were excluded from the study and did not contribute to estimates. In order to understand how this exclusion can impact our estimates we developed a sensitivity analysis.

The aim of the sensitivity analysis presented in the manuscript was to assess potential bias in the reported estimates of the schistosome infection prevalence and the prevalence ratios due to the exclusion of 435 participants with missing UCP-LF CAA test results. To perform the sensitivity analysis, we relied on guidelines provided in “Applying Quantitative Bias Analysis to Epidemiologic Data” by TL. Lash, M P. Fox and A K. Fink, who proposed to assess potential bias in estimates under the range of different hypothetical assumptions.
We considered two hypothetical extreme situations:

1. the best-case scenario, we included an additional 435 samples and assumed that all those samples are negative for the schistosome infection. Under this assumption, we recalculated the prevalence, it was 50.9% (CI 95%: 48.5 - 53.4).
2. The worst-case scenario, we included an additional 435 samples and assumed that all these 435 samples are positive for the schistosome infection. Under this assumption, we re-estimated the prevalence, it was 59.8% (CI 95%: 57.3 - 62.3).

These extreme hypothetical situations provide us information on the maximum and minimum possible values that the prevalence estimates could take if the urine test results were available for all eligible women in our study.
We modified the results section to clarify this point and added the bibliographic reference (21) to justify our methodological approach to performing sensitivity analysis (Lines 265-268)

Line 243: The aPR estimates were similar to ones from the main analysis under both assumptions, with the differences from the original estimates ranging from 0% to 6.3% (less than 10% for all variables) (How did the authors arrive at this range? Not clear. Please include the method used to arrive at this.). There is need for clarity on how the range of differences from the original estimates was calculated.

R: Due to the standardized methodology applied, we included a reference in the methods (ref n 21 line 175). To further clarify with the reviewer, the most common metric used in sensitivity analysis for quantitative bias assessment is a relative change-in-estimate, it is computed as

$$\Delta = \frac{|aPR_S - aPR|}{aPR} * 100\%.$$

where aPR_S stands for the estimate obtained in sensitivity analysis and aPRstands for the original estimate from the main analysis.
The $\Delta > 10\%$ threshold for this metric as an indication of bias is also recommended by TL. Lash, M P. Fox and A K. Fink in “Applying Quantitative Bias Analysis to Epidemiologic Data” and other authors (Hosmer, D. W. , Lemeshow, S. , & Sturdivant, R. X. “Applied logistic regression”). This threshold is widely used in epidemiological studies to assess bias in estimates.

Discussion
Lines 278-279: Additionally, the results of multivariable regression analysis showed that women previously treated with PZQ did not have a different chance to be actively infected with schistosomes. (The authors should include the rationale such as that the treatment with praziquantel does not confer immunity and this shows also that the behavioural or environmental factors persist).

R: Thanks a lot for the comment. The rationale of this statement derives from the fact that MDAs with PZQ are proposed as "preventive chemoprophylactic treatments". we agree that the treatment doesn't confer immunity but it is the entire concept of MDA that encompass the principle that the treatment should decrease the chances of getting re-infected because of more awareness, decreased community prevalence, adoption of protective measures as an example. In this view we think that the result is self-explanatory and adding these considerations would not increase the strength of the statement.

Lines 288-300: The authors did not carry out a comparative test between microscopy and the urine-based upconverting reporter particle, lateral flow (UCP-LF) test detecting circulating anodic antigen to make this type of assertion. They should focus more on the main objective of this study and suggest this aspect as a limitation to the study. A limitation regarding the lack of comparative testing between microscopy and the UCP-LF test should be acknowledged and discussed as even the method used here by the authors has its own challenges.

R: Thanks a lot for the comment. We believe that there is sufficient literature showing that UCP-LF-CAA test is a more sensitive test compared to microscopy hence it has in itself higher likelihood to depict the real prevalence of the infection. We see rather this as a strength of the study, because, normally microscopy is considered as a gold standard mostly due to its cost-effectiveness rather than its performances. In this view, in a prevalence study we don't see it critical not to have microscopy as comparison.

Lines 349-359: Based on the findings of the present study, we strongly advocate for adapting schistosomiasis programs (with the aim of integrating replace with to integrate) preventive schistosomiasis treatment in ANCs of highly endemic contexts. This would contribute to both the WHO and SDGs agenda overall to improving the well-being of women and consequently breaking the vicious cycle of poverty perpetuated by Schistosomiasis. (The authors should capture this in the conclusion section of the abstract).

R: we adapted as requested; we slightly modified the wording in the abstract to avoid redundancy in the information.
