# Supplementary material for: Schistosome infection among pregnant women in the rural highlands of Madagascar: A cross-sectional study calling for public health interventions in vulnerable populations
Source: PLoS Negl Trop Dis. 2024 Apr 16;18(4):e0011766. doi: 10.1371/journal.pntd.0011766 (PMC11051649; doi:10.1371/journal.pntd.0011766)
Supplement: Attachment — Submitted filename: Review PLOS NTD.docx [file pntd.0011766.s004.docx]

**Schistosome infection among pregnant women in the rural highlands of Madagascar: a**

**Cross-sectional study calling for public health interventions in vulnerable populations**

This paper reports a high prevalence of schistosomiasis among a large sample (over 4000) of pregnant women. This is an important topic because data on the prevalence of this neglected tropical disease highlights the need for integration of preventive schistosomiasis treatment into ANC packages in areas where schistosomiasis is highly endemic. I recommend the sharing of these findings with the schistosomiasis and maternal health community.

**General comments**

The current version of the paper reads quite well. However, I would like to make a few suggestions that could further improve the manuscript.

1. The aims and objectives seem to have been swapped, in my opinion. In general a research aim should reflect the broad all-embracing goals of the research project. In other words, the aim should outline the general direction of the research and what it is trying to achieve. The objectives on the other hand should state how the study is going to answer the particular research question. In this case the questions are 1. What is the prevalence of schistosomiasis? 2. What are the factors that are associated with infection? /What are the risk factors for infection?
2. A comparison of the proportions of sociodemographic characteristics among women with and those without test results for schistosomiasis is a good way to dispel fears of underestimating or overestimating prevalence of infection. If the two groups are similar it is highly unlikely that that the estimate would be different even though the missing results were available.

**Specific comments are**

**Line 37**, the first part of the sentence seems incomplete. I suggest rephrasing form “Among 4,448 women aged between 16 and 47 years, the majority of women (70.4%, 38, n = 3,133) resided in rural settings” to “Among 4,448 participating women aged between 16 and 47 years, the majority (70.4%, 38 n = 3,133) resided in rural settings.

**Line 79**. Is the 1 at the end of the sentence the number for a recommendation in WHO document?

**Lines 97 101 (refer to comment # 1 above).**

I suggest rephrasing the aim and objective as follows; “The aim of the study was to provide further evidence to support the adoption of public health strategies targeted at pregnant women for the management and control of schistosomiasis in endemic areas. The objectives of the study were to estimate the prevalence of schistosome infection and identify risk factors for infection among pregnant women in the rural highlands of Madagascar.

**Line 115**. Exclusion criteria does not cover the opposite of inclusion criteria. In other words it should only state who among those that were included were to be excluded. When you say women aged 16 years and above were recruited it automatically means those below 16 were excluded. Exclusion criteria in this case should only include those having a history of congenital anemia, blood transfusion etc. meaning these were excluded from among those that met the inclusion criteria.

**Line 137** Kindly write “LUMC” in full.

**Line 173**. Were 5114 women recruited or that was the total number of women who were seen at the ANC during the study period? By implication using the word “recruited” means these were participants. Kindly rephrase for clarity. I would suggest using the word “screened” in place of “recruited” in line 173 and the chart.
