# Supplementary material for: Schistosome infection among pregnant women in the rural highlands of Madagascar: A cross-sectional study calling for public health interventions in vulnerable populations
Source: PLoS Negl Trop Dis. 2024 Apr 16;18(4):e0011766. doi: 10.1371/journal.pntd.0011766 (PMC11051649; doi:10.1371/journal.pntd.0011766)
Supplement: Attachment — Submitted filename: PNTD-D-23-01372_reviewer Comments 26112023.docx [file pntd.0011766.s005.docx]

**PNTD-D-23-01372_reviewer Comments**

**General Remarks:**

The manuscript titled "Schistosome Infection among Pregnant Women in the Rural Highlands of Madagascar: A Cross-Sectional Study Calling for Public Health Interventions in Vulnerable Populations" makes a commendable effort to address a significant knowledge gap. However, major revisions are recommended to enhance the manuscript's quality and impact by focusing on ensuring accuracy and adherence to best practices in scientific reporting. These considerations aim to contribute to the advancement of knowledge in this critical area. The dedication and commitment reflected in this research are appreciated, and anticipation surrounds the revised version, expecting it to significantly contribute to the scientific discourse in this field.

**Specific Comments:**

1. Species Specificity in Abstract and Methodology: The study lacks specificity regarding the identified Schistosome species in the abstract, methods, results, and discussion sections. Clear differentiation between *S. mansoni* and *S. haematobium*, along with explicit information on the prevalence of each, would strengthen the manuscript.
2. Age Association in Discussion Section: The observed significant association with age requires a more robust justification. Establishing the novelty of this association by drawing comparisons with related studies or providing a more detailed rationale is recommended.
3. Comparison with Control Group: The absence of a comparison with a control group is notable. Including a control group is crucial for a comprehensive interpretation of the observed prevalence among pregnant women. Addressing this deficiency in the study design and analysis is recommended.
4. Methodology Clarity: The methodology could benefit from greater clarity, particularly in providing a step-by-step account of the study procedures. Explicitly stating what was done during the study and ensuring alignment with the presentation of results and subsequent discussions will enhance transparency. The choice of diagnostic method raises a concern, as the POC-CCA test is designed for *S. mansoni,* while the UCP-LF test detects *S. haematobium.* Clarifying the Schistosome species identified and explaining the rationale behind the choice of test would enhance the manuscript.
5. Repetition and Clarity in Abstract: Repetition is observed in the abstract and lines 84-85. Rephrasing to eliminate redundancy will contribute to overall clarity.
6. Title Consideration: Given the need for specificity regarding the identified Schistosome species, consider revising the title to reflect the particular species observed. A more precise title will enhance the manuscript's clarity and relevance.
7. Elaboration on Feasibility and Logistics: The discussion on the feasibility and logistics of implementing preventive measures in Antenatal Care Services could be more detailed. Providing insights into practical integration, addressing potential challenges, and offering implementation process details would strengthen this aspect.
8. Typo in Author Summary (Line 47): A minor typographical error is noted in the Author Summary section (line 47), where "Schistosomiasis" is misspelled as "Schistosomias."
9. Broader Implications for Public Health: Emphasizing the broader implications of your study findings for public health policy and practice is encouraged. Explicitly discussing how your research contributes to shaping policies, interventions, and practices, with a focus on broader public health impact, will enhance the manuscript's significance.

Thank you.
